# Association between PCV and degree of azotemia with serum hepcidin concentration in cats with chronic kidney disease

**DOI:** 10.1093/jvimsj/aalaf010

**Published:** 2026-01-21

**Authors:** Sarah Spencer, Gabriella Lynn, Caroline Wheeler-Jones, Jonathan Elliott

**Affiliations:** Comparative Biological Sciences, Royal Veterinary College, London NW1 0TU, United Kingdom; Royal Veterinary College, London NW1 0TU, United Kingdom; Comparative Biological Sciences, Royal Veterinary College, London NW1 0TU, United Kingdom; Comparative Biological Sciences, Royal Veterinary College, London NW1 0TU, United Kingdom

**Keywords:** inflammation, anemia, iron metabolism, hypoxia, validation

## Abstract

**Background:**

Chronic kidney disease (CKD) in cats is common and often leads to anemia. Hepcidin, an acute-phase protein and regulator of iron metabolism, is a potential biomarker for CKD-associated anemia.

**Hypothesis/Objectives:**

To validate a hepcidin-25 ELISA for cats. To compare serum hepcidin concentrations in cats with CKD grouped by PCV, and explore associations between hepcidin and other clinicopathological variables.

**Animals:**

One hundred client-owned cats with naturally occurring CKD.

**Methods:**

Retrospective cross-sectional study. Cats with azotemic CKD were categorized by PCV: anemic (<28%), low-normal (LN-PCV, 28%-33%), or normal PCV (N-PCV, 35%-43%). LN-PCV and N-PCV groups were matched for plasma creatinine concentration, age, bodyweight, body and muscle condition scores. Associations between serum hepcidin concentrations, serum amyloid A (SAA), and clinicopathological variables were evaluated. Multiple regression analysis identified independent associations with hepcidin.

**Results:**

The hepcidin-25 ELISA demonstrated acceptable precision, reproducibility, and specificity. Hepcidin was higher in anemic cats (median [range], 3.1 ng/mL [1.17-6.58]) compared to LN-PCV (2.21 ng/mL [1.24-6.94]) and N-PCV cats (2.29 ng/mL [1.41-7.72) (*P* = .007), with no difference between the latter two groups (*P* > .99). Hepcidin was weakly positively correlated with plasma creatinine (r = 0.265, *P* = .008) and plasma phosphate (*r* = 0.214, *P* = .034); no significant correlations were observed between hepcidin and PCV, SAA, or other clinicopathological variables. Plasma creatinine alone was associated with hepcidin in multivariable analysis, explaining <15% of the variance.

**Conclusions and clinical importance:**

Serum hepcidin concentration is higher in cats with CKD once anemia is established. Its association with plasma creatinine suggests kidney dysfunction influences its excretion, although additional factors not assessed in this study likely contribute.

## Introduction

Chronic kidney disease (CKD) is a common and important disease affecting predominantly older cats. Non-regenerative anemia is a potential complication that can increase morbidity and is also associated with disease progression and reduced survival.^1^^,^^2^ The pathogenesis of CKD-associated anemia is not well-characterized in cats but is likely multifactorial, involving decreased erythropoietin (EPO) production secondary to loss of functional kidney mass, inflammation-induced iron sequestration, reduced red blood cell lifespan due to uremic toxins, and possibly gastrointestinal hemorrhage.^3^ The level at which to intervene in the treatment of anemia in cats with CKD has not been defined, with International Renal Interest Society (IRIS) guidelines recommending therapy when anemia is affecting quality of life.^4^ There might be value in starting treatment as hematocrit approaches the lower end of the reference interval; however, due to the higher risk of CKD progression and reduced survival associated with PCV at the low end of the reference range.^2^^,^^5^

Serum hepcidin measurement has been proposed as a potential tool for assessing iron metabolism and inflammation in cats with CKD.^6^ Hepcidin, an acute-phase protein (APP), is the most important regulator of body iron absorption. In response to increased hepatic iron stores, hepcidin downregulates ferroportin in the intestinal tract, reducing iron absorption and iron release from macrophages.^7^ Higher circulating concentrations of pro-inflammatory cytokines and reduced renal clearance might lead to elevated hepcidin in CKD.^8^ Hepcidin dysregulation in people with CKD leads to functional iron deficiency and contributes to anemia.^9^^,^^10^ Current evidence in cats with CKD suggests that chronic systemic inflammation leads to increased hepatic hepcidin production and functional iron deficiency, resulting in reduced red cell mass.^6^^,^^11^ A commercially available ELISA is used to measure serum hepcidin concentration in cats, but has not been validated.^6^

This study aimed to validate a commercially available hepcidin-25 assay for use in cats, and to measure serum hepcidin concentrations in cats with CKD, assessing differences between anemic cats, those with low-normal PCV (NL-PCV), and those with normal PCV (N-PCV). Additionally, associations between serum hepcidin concentrations and other clinicopathological variables, including the APP serum amyloid A (SAA), were explored. We hypothesized that anemic CKD cats would exhibit higher hepcidin and SAA concentrations, and that PCV and creatinine would be independently associated with hepcidin levels. Furthermore, we predicted that CKD cats with PCV at the low end of the reference range, but that were not overtly anemic, would have higher serum hepcidin and SAA concentrations compared to those with PCV at the higher end of the reference range, when groups were matched for CKD severity. Should data support activated inflammatory processes and secondary functional iron deficiency in cats with PCV in the lower end of the reference range, there could be a rationale for instigating drug therapy before individuals become overtly anemic, potentially improving outcomes.

## Materials and methods

### Case selection

Medical records of client-owned cats seen at one of two London primary care practices as part of the Royal Veterinary College’s longitudinal CKD and health monitoring program in cats between January 1, 2010, and December 31, 2023, were retrospectively reviewed. Ethical permission for this program has been granted and renewed every five years (2008, 2013, 2018, and 2023). Residual samples were stored and used with informed owner consent. Records were examined for clinical data (eg, signalment, concurrent conditions and medications, clinicopathological results). Azotemic CKD cats were eligible for inclusion. Cats were only included once; when cats were eligible at more than one timepoint, the first visit when a serum sample was available was included in analysis. Azotemic CKD was defined as a plasma creatinine ≥2 mg/dL (≥177 μmol/L) with concurrent urine specific gravity (USG) <1.035, or plasma creatinine concentration ≥2 mg/dL on two consecutive occasions at least two weeks apart without evidence of a pre-renal cause. Cats were subcategorized according to IRIS staging criteria (stages 2-4). Eligible cats were managed with a kidney care diet, although dietary intake was variable.

The exclusion criteria were: concurrent disease that could affect iron metabolism or hepcidin levels, such as infections or neoplasia; conditions that could alter PCV (eg, hematuria, gastrointestinal bleeding, flea burden); cats that were clinically dehydrated or had received intravenous fluid therapy within the proceeding 24-hours; suspected acute kidney injury; previous blood transfusion; hyperthyroidism (total thyroxine > 55 nmol/L) or receiving anti-thyroid medication; cats receiving iron supplementation, darbepoetin or other possible EPO-stimulators (eg, nandrolone).

Cats were categorized into three groups based on PCV. Anemia was defined as PCV < 28%. Cats with PCV within reference range were classified as having either LN-PCV (28%-33% inclusive) or N-PCV (35%-43% inclusive). Where possible, cats with PCV documented more than once in these ranges were chosen to improve reproducibility. Cases in the LN-PCV and N-PCV group were matched for plasma creatinine concentration, age, and duration of feeding a kidney care diet (which also represented time since CKD diagnosis as a kidney care diet was started within four weeks of diagnosis), plus bodyweight, body condition score (BCS), and muscle condition score (MCS). Matching for these characteristics was not possible for the anemic group due to fewer cases being available and the overall presence of more severe CKD, as expected, in anemic cats.

An *a priori* power calculation was undertaken to inform sample size, using mean and SD values for serum hepcidin concentration from a previous study in cats, which utilized the same hepcidin-25 ELISA.^6^ This indicated that 32 cats were required per group to detect a difference in hepcidin concentration of 1.6 ng/mL (SD ±2.3) between groups, with 80% power and 5% type I error rate.

### Blood sampling and laboratory analysis

Blood was collected by jugular venipuncture, or occasionally by cephalic venipuncture, and placed into EDTA, heparinized, and serum blood tubes. Samples were stored on ice for up to five hours before centrifuging at 4 °C for 10 minutes at 3000 rpm. PCV was measured in-house using a micro-hematocrit reader. Routine biochemical variables were measured using heparinized plasma at a commercial veterinary diagnostic laboratory (IDEXX Laboratories, Wetherby, United Kingdom). Serum was aliquoted and stored at −80 °C until SAA and hepcidin measurement. SAA was measured at an external laboratory using an automated assay validated for use in cats (VET-SAA, Eiken Chemical Co., Tokyo, Japan).^12^ Serum hepcidin concentration was measured using a commercially available sandwich ELISA kit validated for use in humans (Hepcidin-25 bioactive ELISA kit, DRG Diagnostics, Marburg, Germany).^6^ Standards were run in triplicate and samples in duplicate in accordance with the manufacturer’s instructions. Duplicates were excluded if coefficient of variation (CV) exceeded > 10%. An equal mix of LN-PCV and N-PCV cats was run on two plates initially, and anemic cats were run on a third plate. Kit standards were used to generate a sigmoidal 4-parameter curve fit using statistical analysis software (GraphPad Prism 10, Boston, United States), and mean hepcidin value for each cat was used in data analysis.

### Serum hepcidin-25 ELISA validation

Validation of the hepcidin ELISA was performed by assessing the assay’s precision, reproducibility, and dilutional parallelism, as described in Supplementary material A.

### Statistical analysis

Statistical analyses were performed using GraphPad Prism 10 statistical software. Statistical significance was set at *P* ≤ .05. Normality of the data was assessed visually and with the Shapiro–Wilk test. Data are presented as mean (SD) for normally distributed data and median (full range) for not normally distributed data. One-way analysis of variance (ANOVA) with Tukey’s multiple comparison test (normally distributed data: age, bodyweight) or Kruskal–Wallis with Dunn’s multiple comparison test (not normally distributed data: plasma hepcidin, plasma creatinine, plasma phosphate, plasma albumin, plasma globulin, USG, BCS, MCS, duration on a kidney care diet) were employed to compare variables between groups (anemic, LN-PCV, N-PCV) and between IRIS stages. Spearman’s correlation was used to assess associations between hepcidin and SAA and other variables. Possible effect of sample storage time on hepcidin concentration was assessed by Spearman’s correlation. SAA was evaluated both as a continuous variable and categorized as follows: <3 ng/mL—not supportive of inflammation, 3-20 ng/mL—inflammation likely (although conditions not typically considered inflammatory can cause such mild elevations), >20 ng/mL—consistent with inflammation. Frequency of cats in each SAA category was compared between PCV group and IRIS stage using Fisher’s exact test.

A multivariable linear regression analysis was conducted to determine independent variables associated with serum hepcidin concentration. The assumptions for multiple linear regression were evaluated, and variables were log-transformed to meet assumptions as necessary. Univariable linear regression analyses were performed using the following variables: PCV, plasma creatinine, phosphate, albumin, globulin, USG, age, bodyweight, and sample storage time. Variables where *P* ≤ .20 were entered into a multivariable model. The final model was derived by manual backward elimination, and goodness-of-fit was evaluated using the adjusted R^2^ value. Residual analysis was performed to check for homoscedasticity and normality, and presence of co-linearity was checked by variance inflation factor. For log-transformed variables, coefficients were back-transformed and are also presented as such.

## Results

### Serum hepcidin-25 ELISA validation

The hepcidin ELISA was shown to have acceptable precision, reproducibility, and specificity, as detailed in Supplementary material A.

Electronic records identified 503 entries with available serum samples from cats with CKD. Of these, 256 met the inclusion criteria. One hundred and sixty-nine cats were excluded due to concurrent disease (hyperthyroidism [*n* = 123], suspected or confirmed infection [*n* = 18], flea burden [*n* = 13], hematuria [*n* = 8], suspected or confirmed neoplasia [*n* = 7]). Fifty-seven cats were excluded due to not eating a kidney care diet, eight due to missing clinical data, five due to nandrolone treatment, three due to clinically detectable dehydration, three due to suspected acute kidney injury, and two due to recent intravenous fluid therapy. After selecting only the first eligible visit per cat, 163 cats remained. Cats were selected for the N-PCV and LN-PCV groups based on matching of the predefined characteristics and constrained by the capacity of two ELISA plates. For the anemic group, cats with the lowest PCVs were selected, limited by the final ELISA plate’s sample capacity. This resulted in 100 cats being included: 37 in the N-PCV group, 31 in the LN-PCV group, and 32 in the anemic group. Cats were mainly domestic shorthair (*n* = 76); other breeds included domestic longhair (*n* = 14), British Shorthair (*n* = 2), Persian crossbreed (*n* = 2), Russian Blue crossbreed (*n* = 2), Siamese crossbreed (*n* = 2), Abyssinian (*n* = 1), and Persian (*n* = 1). Fifty-two cats were female neutered, and 48 cats were male neutered. Twelve cats in the anemic group, four cats in the LN-PCV group, and 13 in the N-PCV group were receiving amlodipine besylate for systemic hypertension. SAA was unavailable in two cats (one in the LN-PCV group and one in the anemic group), due to lack of sample availability.

Clinical and laboratory variables for each group are summarized in Table 1. Mean (SD) PCV in the N-PCV group was 37.4% (2.2), 31.4% (2.3) in the LN-PCV group, and 20.6% (2.7) in the anemic group. Cats in the LN-PCV and N-PCV groups were successfully matched for CKD severity (plasma creatinine concentration), age, bodyweight, BCS, MCS, and duration on a kidney care diet. Anemic cats showed significantly more advanced CKD, with plasma creatinine concentration approximately 1.4-fold higher than the LN-PCV and N-PCV groups and a higher proportion of cats in IRIS stages three and 4. Anemic cats also had significantly lower BCS and MCS than both the LN-PCV and N-PCV groups, and lower bodyweight than N-PCV cats, but age was comparable across groups. Plasma albumin differed between groups (*P* < .0001), with anemic cats having a lower albumin than N-PCV cats (*P* < .0001). USG also differed between groups (*P* = .004), as anemic cats had a significantly lower USG than N-PCV cats (*P* = .003).

**Table 1:** Clinical and laboratory variables in 100 cats with chronic kidney disease with anemia (PCV < 28%), “low-normal” (PCV 28%-33%), or “normal” PCV (PCV 35%-43%). The following variables were matched between low-normal-PCV and normal-PCV groups: plasma creatinine, age, bodyweight, body condition score, muscle condition score, and duration on a kidney care diet. Normally distributed data is displayed as mean (SD); not normally distributed data is displayed as median (range). Differences between groups were assessed using one-way analysis of variance with Tukey’s multiple comparison test (normally distributed data) or Kruskal–Wallis with Dunn’s multiple comparison test (not normally distributed data).

**Variable, units**	**Normal PCV** **(35%**-**43%)**	***N***	**Low-normal PCV** **(28%**-**33%)**	***N***	**Anemic** **(<28%)**	***N***	***P*-value**
PCV, %^*^	37.4 (2.2)	37	31.4 (2.3)	31	20.6 (2.7)	32	ND
**Plasma creatinine (mg/dL) (μmol/L)**	2.36^a^ (1.74-3.58) 209^a^ (154-317)	37	2.43^a^ (1.81-4.87) 215^a^ (160-431)	31	3.30^b^ (1.50-5.87) 292^b^ (133-519)	32	**<.001**
**Plasma phosphate (mmol/L)**	1.26 (0.76-1.85)	37	1.29 (0.81-1.251)	31	1.54 (0.9-3.98)	30	**.050**
**Plasma albumin (g/L)**	31.3^a^ (24.1-35.9)	37	30.3^ab^ (22.1-36.0)	29	28.3^b^ (14.1-38.0)	30	**<.001**
**Plasma globulin (g/L)**	47.0 (36.8-63.0)	37	47.3 (35.3-70.6)	31	45.5 (34.72.2)	30	.700
**Urine specific gravity**	1.017^a^ (1.013-1.028)	21	1.016^ab^ (1.009-1.026)	15	1.015^b^ (1.009-1.034)	20	**.004**
**Age, years**	15.1 (2.64)	37	14.6 (3.33)	31	15.5 (3.68)	32	.609
**Body condition score (1-9)**	5.0^a^ (1.0-7.0)	36	4.5^a^ (2.0-8.0)	30	3.0^b^ (0-7.0)	32	**.003**
**Bodyweight, kg**	4.05^a^ (0.96)	37	3.77^a^ (0.78)	31	3.34^b^ (0.84)	32	**.005**
**Muscle condition score (0-3)**	2.0^a^ (0-3.0)	34	2.0^a^ (0-3.0)	31	1.0^b^ (0-2.0)	31	**<.001**
**IRIS stage (*n*)**	Stage 2 = 34 Stage 3 = 3 Stage 4 = 0	37	Stage 2 = 25 Stage 3 = 6 Stage 4 = 0	31	Stage 2 = 5 Stage 3 = 24 Stage 4 = 3	32	-
**Duration on kidney care diet, days**	162^a^ (28-344)	33	102^a^ (37-392)	28	329^b^ (8-2394)	28	**<.001**
**Serum amyloid A (mg/L)**	2.40 (0-57)	36	2.50 (0-174)	30	1.70 (0-121)	32	.140
**Serum hepcidin concentration (ng/ml)**	2.29^a^ (1.41-7.72)	37	2.21^a^ [1.24-6.94]	31	3.1^b^ [1.17-6.58]	32	**.007**

Overall *P* values are shown and values within a row that have a different superscript letter (^abc^) differ significantly based on *post-hoc* analyses. *P* values ≤ .05 were considered statistically significant. ^*^As PCV was used to pre-define the three groups, statistical analysis was not performed on this parameter. Abbreviation: ND = not determined.

### Acute-phase proteins

**Figure 1:**
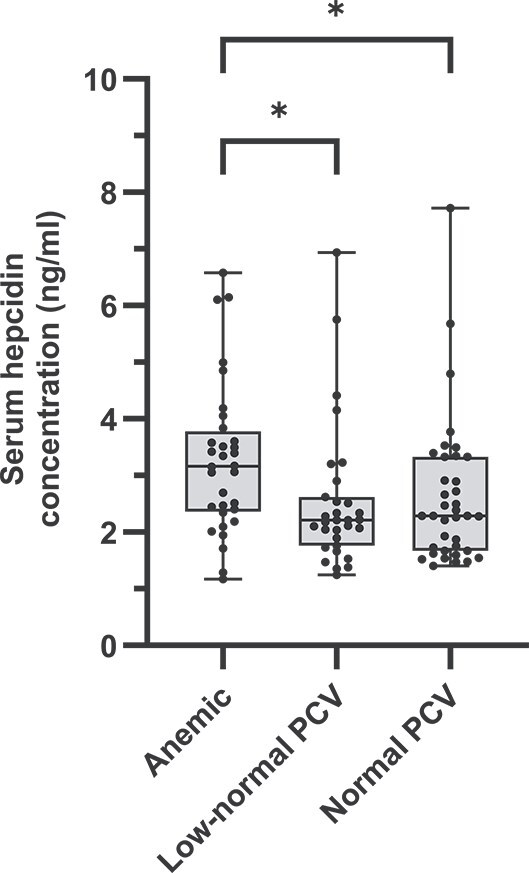
Serum hepcidin concentrations in 100 cats with chronic kidney disease, grouped by PCV (anemic PCV < 25%, low-normal PCV 25%-33%, normal PCV 35%-43%). Graph shows median and range; each point represents an individual cat. ^*^P < .05.

Serum hepcidin concentration was significantly different between groups (*P* = .007), with anemic cats having higher (3.17 ng/mL [1.17-6.58]) levels than both the LN-PCV (2.21 ng/mL [1.24-6.94], *P* = .011) and N-PCV groups (2.29 ng/mL [1.41-7.72], *P* = .037) (Figure 1). Hepcidin did not differ between cats with LN-PCV and N-PCV (*P* > .99). Serum amyloid A did not differ between groups (*P* = .14). Serum hepcidin concentration varied by IRIS stage (*P* = .026), with stage four cats having significantly higher hepcidin than stage two cats (*P* = .041) (Table S1, supplementary material B). Serum amyloid A also differed by IRIS stage (*P* = .039), but post-hoc testing did not reveal significant differences between groups (Table S1, supplementary material B). Seventy-one cats had SAA concentrations not supportive of inflammation (<3 ng/mL), 23 cats had equivocal SAA concentrations (3-20 ng/mL), and four cats had high SAA levels (>20 ng/mL, consistent with inflammation). There was no difference in serum hepcidin concentration between SAA categories (low/normal 2.39 ng/mL [1.29-6.58], moderate 2.51 ng/mL [1.24-6.94], high 2.83 mg/mL [1.17-5.76], *P* = .99). The proportion of cats in each SAA category did not differ between PCV group (*P* = .61) (Table S2, supplementary material B) or IRIS stage (*P* = .75) (Table S3, supplementary material B).

### Correlations between serum hepcidin concentration and clinical variables

**Figure 2:**
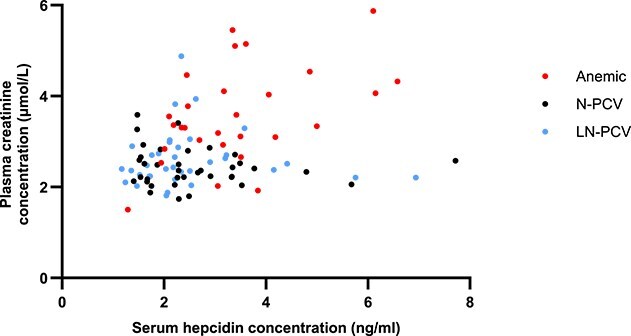
Scatter plot depicting serum hepcidin concentration vs plasma creatinine concentration in 100 cats with chronic kidney disease. Colors represent individual cats’ PCV category (red: anemic [<28%], blue: low normal-PCV [28%-33%], black: normal-PCV [35%-43%]). Spearman’s correlation *r* = 0.265, *P* = .008.

Correlations between serum hepcidin concentration and various clinical variables are shown in Table 2 with plots provided in Supplementary material C. Across the whole cohort, serum hepcidin levels were not significantly associated with PCV (*P* = .15) (Figure S1A, supplementary material C). Hepcidin was significantly weakly positively correlated with plasma creatinine concentration (*r* = 0.265, *P* = .008) (Figure 2) and plasma phosphate concentration (*r* = 0.214, *P* = .034) (Figure S1C, supplementary material C).

**Table 2:** Correlations between serum hepcidin concentration and various clinical variables in 100 cats with chronic kidney disease.

**Variable**	**Spearman r** **(95% CI)**	***P*-value**
**PCV**	−0.146 (−0.338 to 0.0580)	.15
**Plasma creatinine**	**0.265** **(0.0669-0.444)**	**.008**
**Plasma albumin**	0.111 (−0.0951-0.308)	.28
**Plasma globulin**	−0.133 (−0.328-0.0729)	.19
**Plasma phosphate**	**0.214** **(0.0105 to 0.401)**	**.034**
**Serum amyloid A**	−0.0152 (−0.219 to 0.189)	.88
**Age**	0.0782 (−0.126 to 0.276)	.44
**Bodyweight**	−0.0037 (−0.206 to 0.198)	.97
**Urine specific gravity**	0.0704 (−0.198 to 0.335)	.58
**Sample storage time**	0.134 (−0.070-0.327)	.18

Significant correlations (*P* < .05) are denoted in bold. Scatter plots depicting these associations are provided in Supplementary material C.

### Factors associated with serum hepcidin concentration using univariable and multivariable linear regression analyses

All variables, except PCV and age, were log-transformed before linear regression analysis because of non-Gaussian distribution of the data or their residuals. Univariable linear regression was performed between the variables listed in Table 3 and log-hepcidin. Those associated with log-hepcidin at a significance level of *P* < .2 were included in the evaluation of multivariable analysis.; After conducting the model selection process, log-creatinine emerged as the sole independent variable associated with log-hepcidin in the final model (β coefficient 0.240, Exp(β) = 1.271, *P* = .0002). Based on Exp(β) = 1.271, for every doubling of plasma creatinine concentration, serum hepcidin increases by approximately 27.1%. The adjusted R^2^ = 0.136, indicates that just 13.6% of the variance of log-hepcidin was explained by log-creatinine.

**Table 3:** Univariable linear regression analyses to identify predictors of log-serum hepcidin concentration in 100 cats with CKD. Variables where *P* < .20 were entered into a backward-elimination multivariable analysis, where log-creatinine was found to be the only significant independent predictor of log-hepcidin, R^2^ = 0.136, *P* = .0002. For untransformed predictors (age, PCV), the β coefficient reflects the change in log-serum hepcidin per unit increase in the predictor, and exp(β) represents the fold-change in serum hepcidin concentration per unit increase in the original predictor. For log-transformed predictors (bodyweight, plasma albumin, globulin, phosphate, sample storage time, urine specific gravity [USG], and serum amyloid A), β reflects the change in log-serum hepcidin per log-unit change in the predictor (ie, a proportional change), and exp(β) represents the fold-change in hepcidin per fold-change in the variable. Confidence intervals for exp(β) are derived by exponentiating the CI of β.

**Variable**	**Slope** **(β coefficient)**	**95% CI for β**	**Fold change (Exp[β])**	**95% CI for Exp(β)**	**Significance** **(*P*-value)**
**Age**	0.260	1.288,1.808	1.297	0.276,6.098	.74
**Log-Bodyweight**	−0.00300	0.121,0.116	0.997	1.129,1.123	.96
**Log-plasma albumin**	0.0339	0.029,0.0970	1.034	1.029,1.123	.29
**Log-plasma creatinine**	**0.240**	**0.118,0.361**	**1.271**	**1.125,1.435**	**.0002**
**Log-plasma globulin**	**−0.0799**	**0.170,0.0097**	**0.923**	**0.844,0.990**	**.080**
**PCV**	**−3.34**	**6.84,0.152**	**0.035**	**0.001,1.164**	**.061**
**Log-Plasma Phosphate**	**0.162**	**0.023,0.302**	**1.176**	**1.023,1.353**	**.022**
**Log-Sample Storage Time**	**0.161**	**0.042,0.365**	**1.175**	**1.042,1.441**	**.010**
**Log-USG**	**−0.0047**	**−0.0085,0.00086**	**0.995**	**0.992,0.999**	**.119**
**Log-Serum amyloid A**	−0.00150	0.421,0.418	0.999	1.523,1.519	.994

Abbreviation: USG = urine specific gravity.

## Discussion

The hepcidin-25 ELISA, an assay developed for human use, was validated in cats. Serum hepcidin concentrations broadly aligned with a previous study, which used the same assay.^6^ Precision and reproducibility, indicated by low intra- and inter-assay variability, were below accepted thresholds and comparable to the kit’s reported values in human samples.^13^ Recovery was assessed using a dilution series and was acceptable, although lower (78.4%) than the percentage recovery of the kit standard (92.4%); this could indicate that hepcidin levels are slightly underestimated in cat samples using this assay. Ideally, spike-and-recovery testing would have been performed as part of the validation process using recombinant feline hepcidin. Specificity was confirmed by linearity under dilution. A limitation of the dilution series was that only two dilutions were possible as the hepcidin concentration in our cohort was 7.66 ng/mL, and the kit’s lowest limit of detection is 0.153 ng/mL.

The data from the present study demonstrates that anemic cats with CKD exhibit higher circulating hepcidin levels compared to non-anemic CKD cats, implicating hepcidin’s role in limiting iron availability for erythropoiesis in this group of cats. Hepcidin regulates iron absorption and release from stores by binding to ferroportin, leading to its degradation, and reducing iron availability for red cell production.^7^ A previous study found no difference in hepcidin levels between anemic and non-anemic cats with CKD, possibly due to a small number of anemic cats.^6^ This previous study did, however, report an inverse correlation between hepcidin and hematocrit, whereas PCV was not correlated with hepcidin in our study. Discrepant findings could be due to differences in the two study cohorts, including varying therapeutic interventions and the presence of concurrent disease (present in over one-third of the CKD group) in Javard et al.’s study.^6^ As expected, anemic cats in the present study had more severe CKD than non-anemic cats, reflected by higher plasma creatinine concentration and lower USG. As hepcidin is primarily excreted via the kidneys, reduced renal clearance is proposed to partly explain the elevated concentrations in anemic cats.

Indeed, hepcidin correlated positively, though weakly, with plasma creatinine. Furthermore, multivariable analysis revealed plasma creatinine concentration to be the only variable independently associated with hepcidin amongst the variables examined, although it explained little variance in the model, suggesting that other unexamined variables are important. Javard et al.^6^ also reported a positive correlation between circulating creatinine and hepcidin in CKD cats. Human studies have shown conflicting results regarding the relationship between hepcidin and estimated glomerular filtration rate (eGFR). In non-dialysis adult CKD patients, total hepcidin was associated with eGFR, but this was not the case for pediatric patients.^14^ Other studies failed to demonstrate an association between hepcidin-25 (measured with mass spectrometry) and eGFR.^15^^,^^16^ It is proposed that discrepancies result from the use of different assays, particularly the measurement of inactive isoforms in non-specific assays. However, a further two studies showed an association between hepcidin-25 and eGFR, when measured with a specific radioimmunoassay in patients with mild and moderate CKD,^8^ and when measured by mass spectrometry in dialysis patients.^17^ Further research is needed to clarify whether increased hepcidin in anemic CKD cats primarily results from impaired excretion or increased production in response to inflammation or iron status changes. Measurement of urinary hepcidin-25 excretion and GFR in cats would be useful to investigate in future studies.

This study compares hepcidin concentrations in CKD cats, matched for CKD disease severity, between those with LN-PCV (28%-33%) and those with PCV at the higher end of the reference range (35%-43%). No difference in hepcidin was observed, suggesting it does not contribute to within-reference range reductions in red cell mass. Furthermore, SAA did not differ between these groups, suggesting that CKD cats with LN-PCV have not reached the inflammatory threshold required for hepcidin to limit iron availability and therefore erythropoiesis. Given that plasma creatinine concentration was the only independent correlate with hepcidin, the lack of difference in hepcidin between these groups is likely explained by comparable kidney function, ie, plasma creatinine concentration. It would be interesting to investigate other measures of iron metabolism (eg, total iron binding capacity, ferritin) to understand whether cats with LN-PCV experience functional iron deficiency, despite their lack of elevation in serum hepcidin, or whether other factors are driving reduced PCV. Aside from inflammation, hepcidin synthesis is regulated by systemic iron levels, erythropoiesis (particularly the hormone erythroferrone), and hypoxia.^18^^,^^19^

Contrary to our hypotheses, there was no association between serum hepcidin and SAA, and SAA and PCV, implying that inflammation is not a major factor driving increased hepcidin or reduced erythropoiesis in the cohort of CKD cats studied. Furthermore, SAA did not differ between anemic, LN-PCV, and N-PCV groups. Most cats had low SAA concentrations, not supportive of a significant inflammatory state. This is contrary to other reports that have described increased SAA in CKD cats relative to healthy controls, and in anemic CKD cats compared to non-anemic cases.^6^^,^^20^ Confounding results could be due to the presence of concurrent diseases, the use of different assays to measure SAA, and differences in the ages of the cats studied, as APPs can vary with age.^21^ SAA is considered a sensitive marker of inflammation but lacks specificity. It has not been widely studied in chronic inflammatory conditions in cats, and in people, its diagnostic reliability can be reduced when inflammation is chronic.^22^ It is therefore possible that SAA is not an appropriate biomarker of inflammatory status in this group of cats. Anemic cats had lower plasma albumin concentrations than non-anemic cats, potentially reflecting systemic inflammation, or alternatively albumin loss via proteinuria. While CKD in humans is considered a pro-inflammatory state, associations between hepcidin and inflammatory markers, such as CRP, remain inconsistent.^8^^,^^23^ We propose that increased hepcidin in cats with CKD primarily reflects renal excretion rather than a heightened inflammatory state. Measuring additional APPs (eg, fibrinogen, haptoglobin) or cytokines (such as interleukin-6, a direct promoter of hepcidin production) and other aspects of the iron metabolism pathway could clarify this relationship. Additionally, hepcidin can contribute to anemia in CKD, not only through effects on iron metabolism, but also by inhibiting erythroid progenitor proliferation and survival.^24^

The mean PCV in the LN-PCV group was 31.4% (±2.3), just above the < 30% threshold associated with a high risk of CKD progression and mortality in previous studies.^2^^,^^3^ Even minor reductions in red cell mass could exacerbate kidney injury by impairing oxygen delivery to highly metabolically active renal tubular cells and worsening hypoxia secondary to capillary rarefaction,^25^ ultimately resulting in tissue fibrosis. While our data did not implicate hepcidin as a contributor to within-reference range decreases in PCV, further studies should explore the underlying mechanisms by which small PCV reductions might influence CKD progression, and whether interventions before cats become overtly anemic could be beneficial. In people with CKD-associated anemia, hepcidin levels have been reported to decrease after treatment with hypoxia-inducible factor prolyl hydroxylase inhibitors (HIF-PHIs), whereas studies have not consistently shown decreases in hepcidin with EPO treatment.^8^^,^^26–28^ The HIF-PHI molidustat has been investigated in cats,^29^ and the response of hepcidin to treatment in anemic CKD cats warrants further study.

The relationship between inflammation, iron metabolism, and erythropoiesis in CKD is evidently complex, and species variations are expected to exist. While functional iron deficiency (characterized by normal or increased ferritin levels in combination with reduced serum iron and total iron binding capacity) is observed in many cats with CKD, it is not associated with anemia in all cases.^6^^,^^11^ Higher circulating hepcidin concentration is associated with functional iron deficiency, lower hemoglobin, and increased anemia risk in humans, and predicts CKD progression.^9^^,^^30^ This relationship might be limited to individuals with low eGFR, with one study showing no association between hepcidin and hemoglobin in people with higher eGFR.^9^ Hepcidin concentration did not correlate with red blood cell count, hemoglobin concentrations, iron levels, or iron-binding capacities in healthy dogs^31^ and unexpectedly, anemic dogs with CKD demonstrated lower hepcidin levels than non-anemic cases.^32^

Study limitations include the inclusion of only one sample timepoint for each cat. Longitudinal studies are needed to determine whether hepcidin predicts anemia development in non-anemic CKD cats, as has been demonstrated in people.^9^ Complete blood count and blood smear examination were not performed, and therefore other causes for reduced PCV cannot be ruled out, although efforts were made to exclude cats with significant concurrent disease based on history, physical examination, and laboratory results. However, it is possible that some cats did have concurrent disease that could have affected clinicopathological variables, including PCV and SAA. Although the number of cats overall was sizeable and met our *a priori* power calculation, there was a low number of cats with IRIS stage four disease. Anemic cat samples were run on a different plate to the (mixed) LN-PCV and N-PCV samples for the hepcidin ELISA, which could have confounded results due to inter-plate variation. However, given that the mean inter-plate variation was 10.2%, this alone is not expected to explain the significant difference found between this group and the N-PCV and LN-PCV groups.

### Conclusions

Our findings indicate that the main factor associated with serum hepcidin concentration in cats with CKD was plasma creatinine concentration, albeit this explained only a small component of the variability in serum hepcidin concentration. While circulating hepcidin was higher in anemic CKD cats compared to non-anemic cases, it did not differ between non-anemic CKD cats with PCVs at the low and higher end of the reference interval. Evidence for inflammation-driven increases in hepcidin concentration in this group of cats with CKD was lacking.

## Supplementary Material

aalaf010_Supplemental_Files

## Off-label antimicrobial declaration

Authors declare no off-label use of antimicrobials.

## Institutional animal care and use committee or other approval declaration

This study was part of a larger observational cohort for which approval by the Royal Veterinary College’s Ethics and Welfare Committee (URN 2013 1258E) had been granted. Residual samples were stored and used with informed owner consent.

## Abbreviations

ANOVA: analysis of variance
APP: acute phase protein
BCS: body condition score
CI: confidence interval
CV: coefficient of variation
CKD: chronic kidney disease
EPO: erythropoietin
eGFR: estimated glomerular filtration rate
HIF-PFI: hypoxia inducible factor-prolyl hydroxylase inhibitor
IRIS: International Renal Interest Society
LN-PCV: low-normal PCV [group]
MCS: muscle condition score
N-PCV: normal PCV [group]
SAA: serum amyloid A
SD: standard deviation
USG: urine specific gravity
