# Supplementary material for: Association between PCV and degree of azotemia with serum hepcidin concentration in cats with chronic kidney disease
Source: J Vet Intern Med. 2026 Jan 21;40(1):aalaf010. doi: 10.1093/jvimsj/aalaf010 (PMC12881961; doi:10.1093/jvimsj/aalaf010)
Supplement: aalaf010_Supplemental_Files [file aalaf010_supplemental_files.zip › Supplementary_material_A_aalaf010.docx]

**Supplementary material A: Serum hepcidin-25 ELISA validation**

**Methods**

Validation of the hepcidin ELISA was performed by assessing the assay’s precision, reproducibility, and dilutional parallelism. Precision was assessed by calculating the intra-assay CV. ^11^ Three pooled samples were made using four cats each with the lowest, highest and mid-range hepcidin concentrations. Five repeats per pool were measured on the same plate to assess intra-assay variation. The % CV for each sample (low, medium and high hepcidin) was calculated and the average was reported as the intra-assay % CV. Reproducibility was assessed by calculating the inter-assay CV. Duplicates of each of the three pooled samples were run across three different plates on three different days. The % CV for each pooled sample was calculated and the average was reported as the inter-assay % CV. ^11^ Dilutional parallelism was assessed by running a dilution series (neat, 1:2, 1:4) using the sample from the cat with the highest hepcidin concentration diluted with the zero kit standard and comparing with predicted concentrations. A dilution series was also run using the closest corresponding kit standard (9 ng/mL) for comparison.

**Results**

Intra-assay variation was 4.4%, 3.5% and 4.4%, and inter-assay variation was 12.2%, 10.7%, 7.7%, for the pooled low, medium and high samples respectively. No cat’s hepcidin measurement fell outside of the assay's measurable range (0.153 ng/mL - 81 ng/mL) and hepcidin values occurred on the linear portion of the standard curve. There was no correlation between sample storage time and serum hepcidin concentration (r=.134, P=.18). The intra- and inter-assay variation was such that a difference in serum hepcidin concentration of 1.6 ng/ml between groups – used for power calculation and sample size determination – was measurable.

The recovery percentages for the dilution series performed on the cat with the highest hepcidin concentration (mean 7.66 ng/mL) was 84.2% (1:2 dilution) and 72.5% (1:4 dilution), giving a mean recovery of 78.4%. A dilution series using the 9 ng/mL kit standard gave a mean recovery of 92.4%.
