# Supplementary material for: Association between PCV and degree of azotemia with serum hepcidin concentration in cats with chronic kidney disease
Source: J Vet Intern Med. 2026 Jan 21;40(1):aalaf010. doi: 10.1093/jvimsj/aalaf010 (PMC12881961; doi:10.1093/jvimsj/aalaf010)
Supplement: aalaf010_Supplemental_Files [file aalaf010_supplemental_files.zip › Supp_material_B_aalaf010.docx]

**Supplementary material B**

Table S1: PCV, serum hepcidin concentration and serum amyloid concentration in 100 cats with chronic kidney disease categorized according to International Renal Interest Society stage (2-4). Values within a row that have a different superscript letter (^abc^) differ significantly based on *post-hoc* analyses. *Serum amyloid A was significantly different between IRIS stage (P=.039) but *post-hoc* tests did not reveal where this difference lay between groups.

| **Variable** | **IRIS stage 2** | **N** | **IRIS stage 3** | **N** | **IRIS stage 4** | **N** |
| --- | --- | --- | --- | --- | --- | --- |
| PCV (%) | 33.6^a^(5.34) | 63 | 24.9^b^  (6.98) | 33 | 19.3^b^  (2.50) | 4 |
| Serum hepcidin concentration (ng/mL) | 2.29^a^  (1.17-7.72) | 63 | 2.47  (1.38-6.58) | 33 | 3.50^b^  (3.34-6.10) | 4 |
| Serum amyloid A concentration (mg/L)* | 2.50  (0-174) | 61 | 1.80  (0-121) | 33 | 1.15  (0.4-2.2) | 4 |

Table S2: Frequency table of 98 cats with CKD categorized by serum amyloid A (SAA) and PCV group. SAA categorization was as follows: Low - <3 ng/ml, not supportive of inflammation; medium/equivocal - 3-20 ng/ml; high - >20ng/ml, consistent with inflammation. There was no significant difference between PCV groups, analysed by Fisher’s exact test (P=.61).

|  | **Low SAA**  **(<3 ng/ml)** | **Medium/equivocal SAA (3-20 ng/ml)** | **High SAA**  **(3-20 ng/ml)** | **Total no. of cats** |
| --- | --- | --- | --- | --- |
| Anemic (PCV<28%) | 24 | 6 | 2 | 32 |
| Low-normal PCV (28-33%) | 19 | 10 | 1 | 30 |
| Normal PCV  (35-43%) | 28 | 7 | 1 | 36 |
| Total no. of cats | 71 | 23 | 4 | 98 |

Table S3: Frequency table of 98 cats with CKD categorized by serum amyloid A (SAA) and International Renal Interest Society stage (2-4). SAA categorization was as follows: Low - <3 ng/ml, not supportive of inflammation; medium/equivocal - 3-20 ng/ml; high - >20ng/ml, consistent with inflammation. There was no difference between IRIS stages, analysed by Fisher’s exact test (P=.75).

|  | **Low SAA**  **(<3 ng/ml)** | **Medium/equivocal SAA (3-20 ng/ml)** | **High SAA**  **(3-20 ng/ml)** | **Total no. of cats** |
| --- | --- | --- | --- | --- |
| IRIS stage 2 | 43 | 16 | 2 | 61 |
| IRIS stage 3 | 24 | 7 | 2 | 33 |
| IRIS stage 4 | 4 | 0 | 0 | 4 |
| Total no. of cats | 71 | 23 | 4 | 98 |
