# Supplementary material for: Association between PCV and degree of azotemia with serum hepcidin concentration in cats with chronic kidney disease
Source: J Vet Intern Med. 2026 Jan 21;40(1):aalaf010. doi: 10.1093/jvimsj/aalaf010 (PMC12881961; doi:10.1093/jvimsj/aalaf010)
Supplement: aalaf010_Supplemental_Files [file aalaf010_supplemental_files.zip › Supp_material_C_aalaf010.docx]

Figures S1 A-I. Scatter plots showing serum hepcidin concentration versus various clinical variables in 100 cats with chronic kidney disease. Colors represent individual cats’ PCV category (red: anemic [<28%]: blue: low normal-PCV [28-33%], black: normal-PCV [35-43%]). Spearman’s correlation (r) and P values are shown on each graph.
